# Systematically analysis of decompensated cirrhotic patients with spontaneous bacterial peritonitis to identify diagnostic and prognostic indexes

**DOI:** 10.1186/s12879-023-08731-w

**Published:** 2023-11-11

**Authors:** Tao Du, Qing-ping Li, Gui-xiang Jiang, Hui-yuan Tan, Jiao-hua Wu, Shan-yu Qin, Bing Yu, Hai-xing Jiang, Wei Luo

**Affiliations:** 1 Department of Gastroenterology, the First Affiliated Hospital of Guangxi Medical University, No. 6, Shuangyong Road, Nanning, 530021 China; 2 Department of Gastroenterology, the Second Affiliated Hospital of Guangxi Medical University, Nanning, China

**Keywords:** Cirrhosis, Spontaneous bacterial peritonitis, Laboratory test indexes, Predictive value, Prognosis analysis

## Abstract

**Background:**

Spontaneous bacterial peritonitis (SBP) is a common complication in patients with cirrhosis. The diagnosis of SBP is still mostly based on ascites cultures and absolute ascites polymorphonuclear (PMN) cell count, which restricts the widely application in clinical settings. This study aimed to identify reliable and easy-to-use biomarkers for both diagnosis and prognosis of cirrhotic patients with SBP.

**Methods:**

We conducted a retrospective study including 413 cirrhotic patients from March 2013 to July 2022 in the First Affiliated Hospital of Guangxi Medical University. Patients’ clinical characteristics and laboratory indices were collected and analyzed. Two machine learning methods (Xgboost and LASSO algorithms) and a logistic regression analysis were adopted to screen and validate the indices associated with the risk of SBP. A predictive model was constructed and validated using the estimated area under curve (AUC). The indices related to the survival of cirrhotic patients were also analyzed.

**Results:**

A total of 413 cirrhotic patients were enrolled in the study, of whom 329 were decompensated and 84 were compensated. 52 patients complicated and patients with SBP had a poorer Child–Pugh score (*P* < 0.05). Patients with SBP had a greater proportion of malignancies than those without SBP(*P* < 0.05). The majority of laboratory test indicators differed significantly between patients with and without SBP (*P* < 0.05). Albumin, neutrophil-to-lymphocyte ratio (NLR), and ferritin-to-neutrophil ratio (FNR) were found to be independently associated with SBP in decompensated cirrhotic patients using LASSO algorithms, and logistic regression analysis. The model established by the three indices showed a high predictive value with an AUC of 0.808. Furthermore, increased neutrophils, ALP, and C-reactive protein-to-albumin ratio (CAR) were associated with the shorter survival time of patients with decompensated cirrhosis, and the combination of these indices showed a greater predictive value for cirrhotic patients.

**Conclusions:**

The present study identified FNR as a novel index in the diagnosis of SBP in decompensated patients with cirrhosis. A model based on neutrophils, ALP and CAR showed high performance in predicting the prognosis of patients with decompensated cirrhosis.

**Supplementary Information:**

The online version contains supplementary material available at 10.1186/s12879-023-08731-w.

## Introduction

Spontaneous bacterial peritonitis (SBP) refers to peritoneal bacterial infection with no apparent source of intra-abdominal infection [1, 2]. SBP is a common complication in patients with cirrhosis, with an estimated frequency of 10–30%, and the outcome of patients with SBP remains unfavorable despite the advancement in therapy [3, 4]. Multiple mechanisms have been shown to contribute to the occurrence of SBP in cirrhosis, including impaired humoral and cell-specific immunity caused by hypoalbuminemia [5], increased intestinal permeability caused by portal hypertension, and the impaired neutrophil and reticuloendothelial system [6, 7]. All of these factors lead to an increased susceptibility to infection.

Recently, novel indices have been proven to be potential biomarkers of SBP in cirrhosis. For example, the general laboratory test indices of ascites calprotectin and lactoferrin [8], serum procalcitonin and C-reactive protein (CRP) [9], platelet and neutrophil-to-lymphocyte ratio (NLR) [10, 11] have been documented in the diagnosis of SBP in cirrhosis. In addition, ascites endocane [12], IL-17 [13] and CD206 [14] have also been reported as biomarker candidates for the diagnosis of SBP in cirrhosis. However, these results have yet to be validated due to limitations such as sample size and confounding factors. Therefore, it remains necessary to find reliable biomarkers for the diagnosis and prognosis of cirrhotic patients with SBP.

In this study, we retrospectively analyzed the data from cirrhotic patients aiming to examine the blood biomarkers associated with SBP and constructed a model to diagnose SBP in decompensated cirrhosis. We also included patients with compensated cirrhosis because SBP was the important symbol indicating that patients with compensated cirrhosis would develop into the decompensated period. We compared the differences in indicators between compensated and decompensated cirrhosis in order to find out the characteristics of disease progression to prevent the occurrence of SBP in the early stage. Finally, we attempted to identify the blood biomarkers associated with the survival of decompensated cirrhotic patients. Our results would shed light on the crucial role of blood biomarkers in the diagnostic and prognostic value of liver cirrhosis with SBP.

## Materials and methods

### Data collection

We retrospectively enrolled cirrhotic patients being hospitalized at the First Affiliated Hospital of Guangxi Medical University between March 2013 and July 2022. The inclusion criteria were as follows: (1) age over 18 years; (2) the diagnosis of liver cirrhosis with or without SBP was made by clinical, laboratory, or imaging tests; (3) availability of complete clinicopathological and follow-up data after discharge. Exclusion criteria were pregnancy, secondary peritonitis, peritoneal dialysis-associated peritonitis, and chronic liver disease without cirrhosis. The Child–Pugh classification was determined as following factors: (1) encephalopathy; (2) ascites; (3) serum bilirubin level; (4) serum albumin level; (5) prothrombin time.

The diagnosis of SBP was made based on the 2017 Chinese Guidelines for the management of ascites and its related complications in cirrhosis [15]. The clinical diagnosis of SBP should be considered if patients with cirrhosis are in the following conditions: (1) One or more of the following symptoms or signs occur: fever, abdominal pain, abdominal tenderness or rebound tenderness, refractory ascites, etc.; (2) One or more of the following results on laboratory tests are positive: ascites bacteria culture, absolute ascites PMN cell counts ≥ 0.25 × 10^9^/L, and serum procalcitonin (PCT) > 0.5 ng/ml, etc. (3) Infection of other sites is excluded.

### Clinical data collection and definitions

Clinicopathologic features and laboratory data were collected from the patient's electronic medical records, and laboratory data within two days of hospital admission for diagnosing SBP. Clinicopathologic data of cirrhosis included patients' age, sex, Child–Pugh score, cancers, infection status, and laboratory test indices. We selected only the laboratory test indices that were tested for the first time after the diagnosis of cirrhosis. The index was calculated as a formula: NLR = neutrophil count/ lymphocyte count ratio; PLR = platelet count/ neutrophil count ratio; FAR = ferritin levels/ albumin levels ratio; FNR = ferritin levels/ neutrophil count ratio; CAR = C-reactive protein level/ albumin level ratio.

### Follow-up

All the patients were followed for a minimum of one month after recruitment, and thereafter every three to six months at the discretion of the attending physician. The last follow-up was on December 31, 2022.

### Statistical analysis

Normal continuous data are presented as mean standard deviation and compared to Student's t-test, while nonnormal data are presented as the median and interquartile range (IQR) and compared to Mann–Whitney's U-test. Categorical data are presented as absolute numbers (percent) and were compared using the chi-square test or Fisher's exact test for small samples. Xgboost algorithms and LASSO algorithms were used to verify key features related to the SBP. Logistic regression and Cox regression analysis were applied to identify key features related to the SBP and the constructed predictive model. The predicted value was calculated using receiver operating characteristic (ROC) and area under the curve (AUC) analyses. A Kaplan–Meier plot and log-rank test were used to analyze survival data using Kaplan–Meier plots, which were visualized by the “survminer” R package [16]. The risk score was calculated by multiplying the value and the coefficients of each index from the Cox regression model according to the previously described [17]. Statistical significance was defined as a P-value less than 0.05. All analyses were performed with the R software (version 4.2.2).

## Results

### Clinical characteristics of included data

Data were collected from a total of 413 cirrhotic patients with an average age of 54.4 to 12.3 years, including 336 male and 77 female patients. 329 were decompensated, while 84 were compensated. The etiology of cirrhosis included HBV infection (348 patients) and others (65 patients). 52 (12.6%) patients complicated SBP, 188 patients complicated cancer, the majority of which were HCC (175 patients). The laboratory tests revealed that the inflammatory indices, liver function indices, and nutritional indices were significantly different between compensated and decompensated patients (*P* < 0.05). The median follow-up time was eight months, with a range of one month to ninety-four months. The details of the clinical features were given in Table 1. And we also provided an additional table file showing the details of laboratory tests and clinical characteristics in patients with SBP (see Additional file 1).

**Table 1:** Clinical characteristics of included data

Features	Total	Compensated (*N* = 84)	Decompensated (*N* = 329)	*P* value
Age (years)	54.4 ± 12.3	54.5 ± 11.8	54.3 ± 12.5	0.911
Sex				0.498
Male	336 (81.4%)	71 (84.5%)	265 (80.5%)	
Female	77 (18.6%)	13 (15.5%)	64 (19.5%)	
Etiology				1.000
HBV	348 (84.3%)	71 (84.5%)	277 (84.2%)	
Others	65 (15.7%)	13 (15.5%)	52 (15.8%)	
Class				< 0.001
Child–Pugh A	154 (37.3%)	84 (100%)	70 (21.3%)	
Child–Pugh B	168 (40.7%)	0 (0%)	168 (51.1%)	
Child–Pugh C	91 (22.0%)	0 (0%)	91 (27.7%)	
SBP				< 0.001
Yes	52 (12.6%)	0 (0%)	52 (15.8%)	
No	361 (87.4%)	84 (100%)	277 (84.2%)	
Cancers	188 (45.5%)	42 (50%)	146 (44.4%)	0.423
WBC (× 10^9^/L)	6.2 (4.4–8.1)	6.4 (4.8–7.8)	6.2 (4.3–8.3)	0.725
Neutrophil (× 10^9^/L)	3.8 (2.4–5.6)	3.7 (2.2–5.2)	3.8 (2.5–5.7)	0.404
Lymphocyte (× 10^9^/L)	1.2 (0.8–1.6)	1.5 (1.1–2.0)	1.1 (0.8–1.5)	< 0.001
Platelet (× 10^9^/L)	131.6 (83.6–206.2)	179.8 (110.9–223.3)	122.0 (76.4–184.0)	< 0.001
TBil (μmol/L)	25.6 (15.0–57.1)	14.1 (9.4–19.2)	33.4 (17.4–68.6)	< 0.001
ALB (g/L)	30.9 (26.3–35.5)	36.5 ± 5.2	29.7 ± 6.2	< 0.001
AST (U/L)	67.0 (39.0–130.0)	40.5 (28.5–69.0)	79.0 (44.0–148.0)	< 0.001
ALT (U/L)	42.0 (25.0–72.0)	31.0 (20.0–49.5)	44.0 (27.0–86.0)	< 0.001
ALP (U/L)	138.0 (97.0–203.0)	112.0 (84.0–171.5)	147.0 (101.0–213.0)	0.002
CRP (mg/L)	12.3 (3.8–37.0)	5.0 (1.7–12.6)	16.6 (5.3–40.2)	< 0.001
Ferritin (ng/ml)	491.7 (143.9–1014.6)	417.8 (158.8–731.1)	555.3 (136.6–1123.5)	0.089
FAR	15.5 (4.8–34.7)	10.7 (4.5–20.0)	17.9 (4.9–42.1)	0.004
FNR	121.2 (40.0–306.1)	104.0 (36.2–253.8)	127.6 (41.7–318.9)	0.367
PLR	108.8 (75.8–172.5)	119.1 (83.9–166.2)	107.5 (72.2–173.6)	0.393
NLR	3.2 (2.1–5.2)	2.6 (1.6–4.1)	3.4 (2.2–5.4)	< 0.001
CAR	0.4 (0.1–1.3)	0.1 (0.0–0.4)	0.6 (0.2–1.5)	< 0.001

### Comparison of decompensated cirrhotic patient complicated with and without SBP

The decompensated cirrhosis data were divided into two groups based on whether the patients complicating SBP. As shown in Table 2, we found that patients with SBP had a poorer Child–Pugh score (*P* < 0.05) and the proportion of cancers was higher in patients with SBP than in patients without SBP (*P* < 0.05). Regarding the laboratory tests, the results showed that most of the inflammatory indices, liver function indices and nutritional indices were significantly different between patients with and without SBP, except lymphocytes, platelets, ALP and PLR (*P* < 0.05).

**Table 2:** Comparison of decompensated cirrhotic patients complicated with and without SBP

Features	Non-SBP (*N* = 277)	SBP (*N* = 52)	Chi-square value	*P* value
Age (years)	54.6 ± 12.3	52.9 ± 13.4		0.379
Sex	58 (20.9%)	6 (11.5%)		0.167
Male	219 (79.1%)	46 (88.5%)		
Female				
Etiology	229 (82.7%)	48 (92.3%)		0.123
HBV	48 (17.3%)	4 (7.7%)		
Others				
Class			78.457	< 0.001
Child–Pugh A	70 (25.3%)	0 (0%)		
Child–Pugh B	151 (54.5%)	17 (32.7%)		
Child–Pugh C	56 (20.2%)	35 (67.3%)		
Cancers	144 (52%)	39 (75%)		0.004
WBC (× 10^9^/L)	6.0 (4.2–7.8)	7.5 (4.8–11.0)		0.003
Neutrophil (× 10^9^/L)	3.6 (2.3–5.3)	5.2 (2.9–8.2)		0.008
Lymphocyte (× 10^9^/L)	1.1 (0.8–1.5)	1.0 (0.8–1.4)		0.317
Platelet (× 10^9^/L)	125.0 (77.9–200.1)	107.0 (70.7–156.6)		0.133
TBil (μmol/L)	30.5 (16.8–57.9)	66.8 (33.4–232.4)		< 0.001
ALB (g/L)	30.5 ± 6.1	25.8 ± 5.0		< 0.001
AST (U/L)	72.0 (43.0–144.0)	94.0 (59.0–259.0)		0.005
ALT (U/L)	43.0 (26.0–81.0)	47.5 (33.0–137.5)		0.022
ALP (U/L)	150.0 (100.0–222.0)	138.0 (111.5–176.0)		0.207
CRP (mg/L)	14.3 (4.6–37.7)	27.6 (11.5–78.1)		< 0.001
Ferritin (ng/ml)	452.2 (123.2–997.8)	1021.3 (478.2–3262.3)		< 0.001
FAR	15.2 (4.1–33.5)	45.0 (18.1–117.8)		< 0.001
FNR	116.3 (37.6–262.4)	312.5 (84.0–622.5)		< 0.001
PLR	108.7 (75.8–176.8)	97.6 (58.8–170.5)		0.379
NLR	3.3 (2.2–5.2)	4.8 (2.4–10.2)		0.013
CAR	0.5 (0.1–1.3)	1.0 (0.5–3.2)		< 0.001

### Effect of cancers on decompensated cirrhotic patients with SBP

To investigate the effect of cancer on cirrhosis with SBP, we performed a subgroup analysis for decompensated cirrhotic patients with SBP who had cancer or not. As shown in Table 3, the indices of laboratory tests of patients with SBP were strongly influenced by the cancer in terms of WBC, Neutrophil, TBil, CRP, FNR, PLR, NLR and CAR. In the patients without SBP, more laboratory test indices were affected by cancer, but the effect differed between those with and without SBP in terms of lymphocytes, platelets, TBil, AST, ALT, ALP, ferritin, FAR and NLR, suggesting that cancer had a differential effect on these indices in decompensated cirrhotic patients with SBP.

**Table 3:** Effect of cancers on decompensated cirrhosis with and without SBP

Features	Without SBP (*N* = 277)			With SBP (*N* = 52)		
	Cancer (*N* = 133)	No cancer (*N* = 144)	*P* value	Cancer (*N* = 13)	No cancer (*N* = 39)	*P* value
WBC (× 10^9^/L)	6.5 (5.1–8.9)	5.0 (3.8–7.2)	< 0.001	11.0 (7.5–13.6)	6.6 (4.6–8.9)	0.045
Neu (× 10^9^/L)	4.1 (3.0–6.2)	3.2 (2.1–4.9)	< 0.001	8.9 (5.7–11.6)	3.9 (2.8–6.8)	0.012
Lymphocyte (× 10^9^/L)	1.2 (0.9–1.5)	1.0 (0.7–1.5)	0.005	1.0 (0.6–1.1)	1.2 (0.8–1.4)	0.176
Platelet (× 10^9^/L)	163.2 (112.6–244.6)	90.0 (62.9–147.6)	< 0.001	136.3 (104.3–172.0)	102.9 (70.7–143.7)	0.078
TBil (μmol/L)	32.2 (18.2–57.5)	26.1 (15.0–63.2)	0.329	39.2 (19.4–98.0)	90.1 (45.2–279.4)	0.018
ALB (g/L)	30.9 ± 5.4	30.1 ± 6.7	0.292	24.4 (21.6–28.7)	25.3 (22.1–28.4)	0.634
AST (U/L)	104.0 (65.0–174.0)	48.5 (30.5–91.0)	< 0.001	81.0 (64.0–127.0)	98.0 (59.0–310.5)	0.627
ALT (U/L)	57.0 (34.0–92.0)	33.0 (24.0–60.5)	< 0.001	47.0 (34.0–84.0)	59.0 (30.5–236.0)	0.612
ALP (U/L)	179.0 (126.0–278.0)	126.0 (89.0–180.5)	< 0.001	163.0 (111.0–184.0)	126.0 (114.0–166.5)	0.479
CRP (mg/L)	24.1 (9.4–46.9)	8.2 (2.7–25.2)	< 0.001	89.2 (48.1–131.8)	19.0 (10.8–41.7)	0.009
Ferritin (ng/ml)	663.1 (238.7–1322.2)	335.8 (64.6–792.5)	< 0.001	579.8 (221.2–2876.3)	1187.8 (654.5–3537.5)	0.276
FAR	19.5 (8.4–37.4)	11.5 (2.3–31.1)	< 0.001	27.2 (10.9–112.8)	51.6 (24.9–130.1)	0.304
FNR	145.8 (66.5–262.4)	88.3 (24.8–263.2)	0.008	101.2 (59.2–349.0)	415.8 (93.3–737.0)	0.035
PLR	138.1 (88.4–198.8)	92.7 (64.7–145.3)	< 0.001	118.0 (93.5–293.8)	91.4 (53.7–144.7)	0.044
NLR	3.5 (2.4–5.1)	3.2 (1.9–5.3)	0.241	7.7 (5.9–14.5)	3.4 (1.9–7.1)	0.006
CAR	0.8 (0.3–1.6)	0.3 (0.1–0.8)	< 0.001	4.4 (2.0–5.3)	0.8 (0.5–1.6)	0.011

### Screening risk factors associated with SBP in decompensated cirrhotic patients

**Fig. 1:**
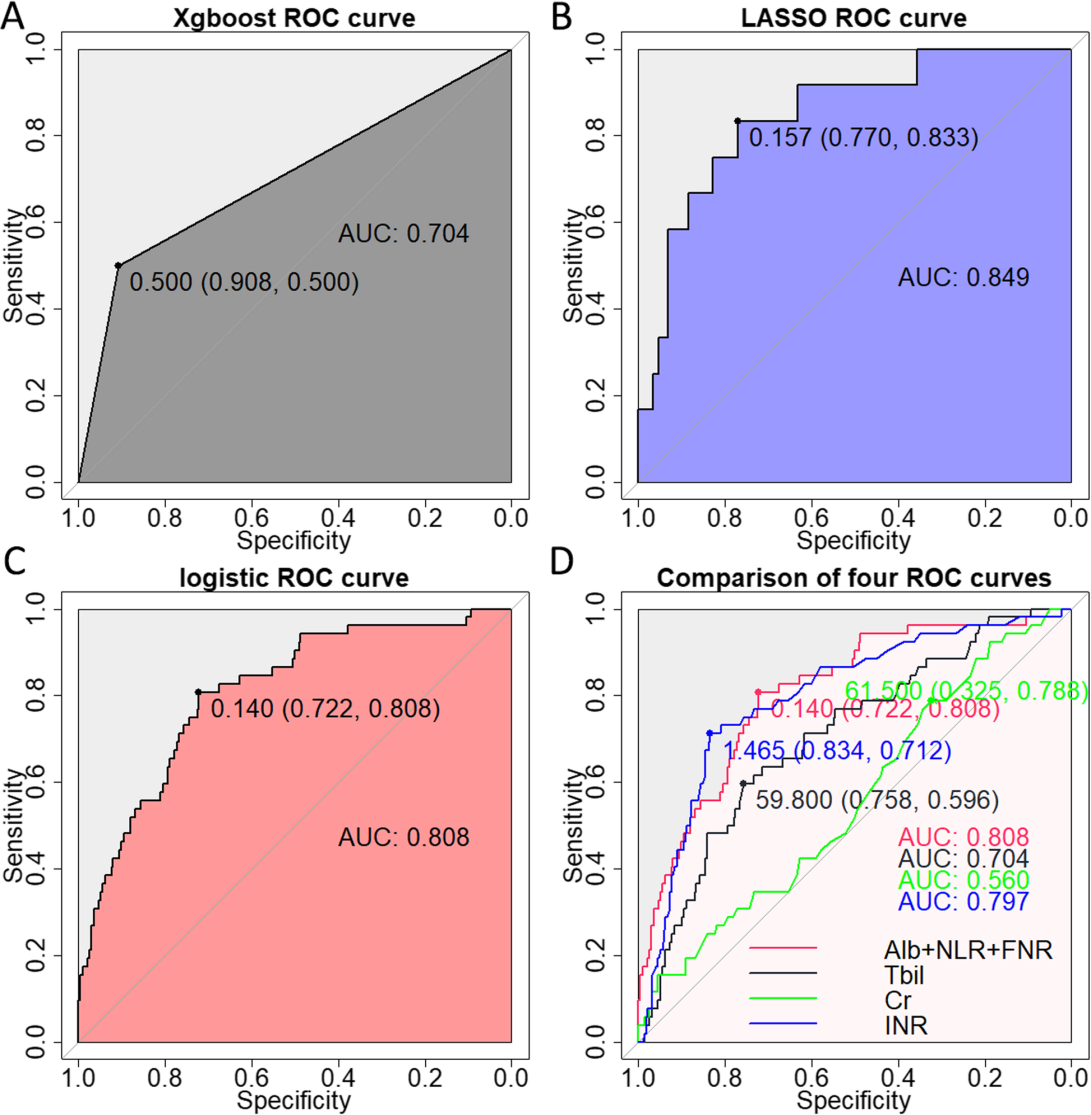
Identify risk factors associated with SBP in cirrhotic patients. Predictive value of **A** Xgboost algorithms **B** LASSO algorithms for SBP using the clinical features **C** logistic regression model for SBP using the indexes from overlapped of Xgboost algorithms and LASSO algorithms results. **D** ROC curves for the creatinine, INR and total bilirubin in predicting SBP

To determine the laboratory test indices related to SBP in decompensated cirrhotic patients, two machine learning methods, Xgboost and LASSO algorithms, were adopted to study the risk factors related to SBP in decompensated cirrhotic patients. The Xgboost and LASSO algorithms identified 10 and 14 indices related to SBP risk with an AUC value of 0.704 and 0.849, respectively (Fig. 1A, B). Then we overlapped these indices and identified six common indices including Neutrophils, Alb, ALP, NLR, CAR, FNR. Then we included these common indices in the multivariate logistic regression model and found that only ALB, NLR and FNR were independently associated with the SBP in decompensated cirrhotic patients (Table 4). We used these three indices to create a diagnostic model and found that the diagnostic value was high, with an AUC value of 0.808 (Fig. 1C). In addition, we also compared the discrimination of the model with other indicators via AUC value, including creatinine, INR and total bilirubin, which were individual components of MELD score, and the results showed that our model had the highest AUC value compared to these indices (Fig. 1D).

**Table 4:** Logistics regression for the index associated with SBP in cirrhotic patients

Features	OR (univariable)	*P* value	OR (multivariable)	*P* value
ALB	0.87 (0.82–0.92)	< 0.001	0.88 (0.83–0.94)	< 0.001
ALP	1.00 (0.99–1.00)	0.050	1.04 (0.96–1.12)	0. 309
CAR	1.43 (1.22–1.67)	< 0.001	1.16 (0.91–1.48)	0..218
Neu	1.17 (1.08–1.28)	< 0.001	1.10 (0.96–1.26)	0 .183
NLR	1.11 (1.05–1.16)	< 0.001	0.99 (0.99–1.00)	0.019
FNR	1.00 (1.00–1.00)	< 0.001	1.00 (1.00–1.00)	< 0.001

The false positive rate and false negative rate of the diagnostic model were 2.53% and 80.77%, the high false negative rate indicated that the model’s role in diagnosing SBP was rather limited. However, this model might be an ideal exclusive diagnostic tool due to its extremely low false positive rate.

### Identify risk factors related to the prognosis of decompensated cirrhotic patients

**Fig. 2:**
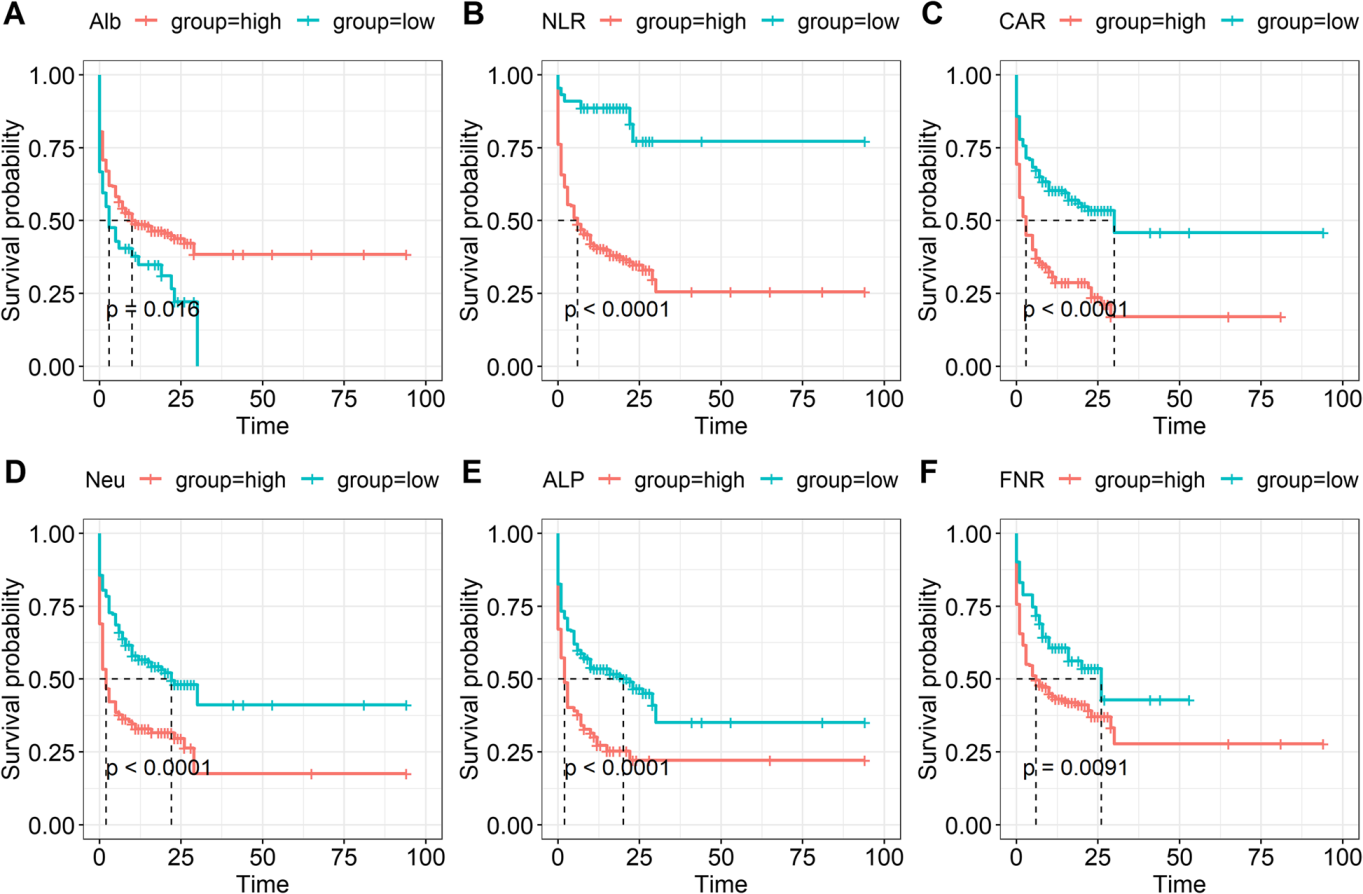
Kaplan–Meier plots of the indices with the decompensated cirrhotic patients. **A** Alb; **B** NLR; **C** CAR; **D** neutrophils; **E** ALP; **F** FNR

**Fig. 3:**
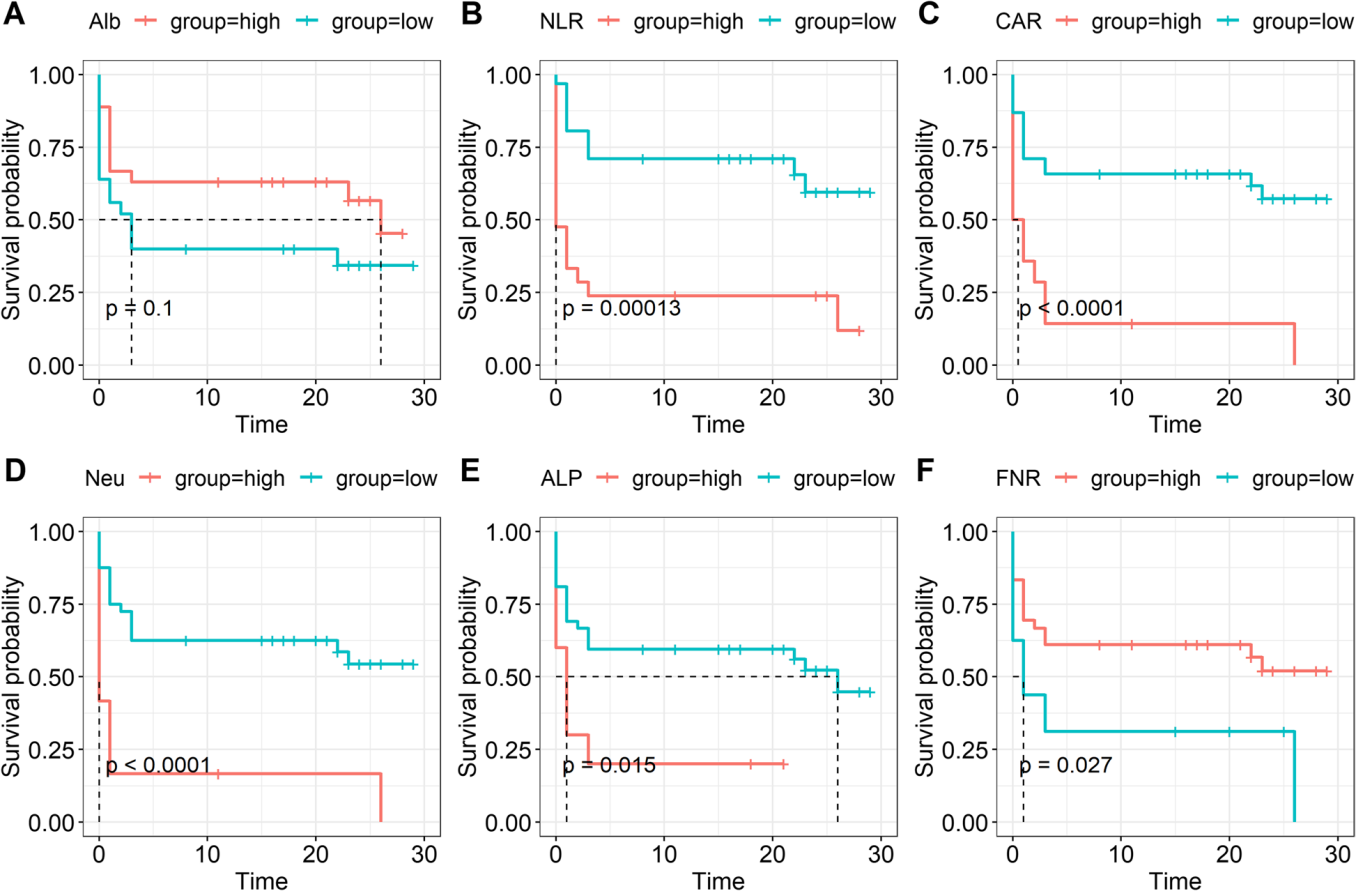
Kaplan–Meier plots of the indices with the decompensated cirrhotic patients with SBP. **A** Alb; **B** NLR; **C** CAR; **D** neutrophils; **E** ALP; **F** FNR

We first estimated the association of the eight indices with the survival of decompensated cirrhotic patients, using the optimal cut-off value obtained by the “survminer” R package, we found that all six indices neutrophils, ALB, ALP, NLR, CAR, and FNR were associated with survival of decompensated patients with cirrhosis and high neutrophil levels with a shorter survival time, while ALB was associated with a longer survival time (*P* < 0.05; Fig. 2). However, when focusing on the 52 SBP patients, the results did not show that Alb and ALT were associated with survival of SBP patients (*P* > 0.05; Fig. 3).

**Fig. 4:**
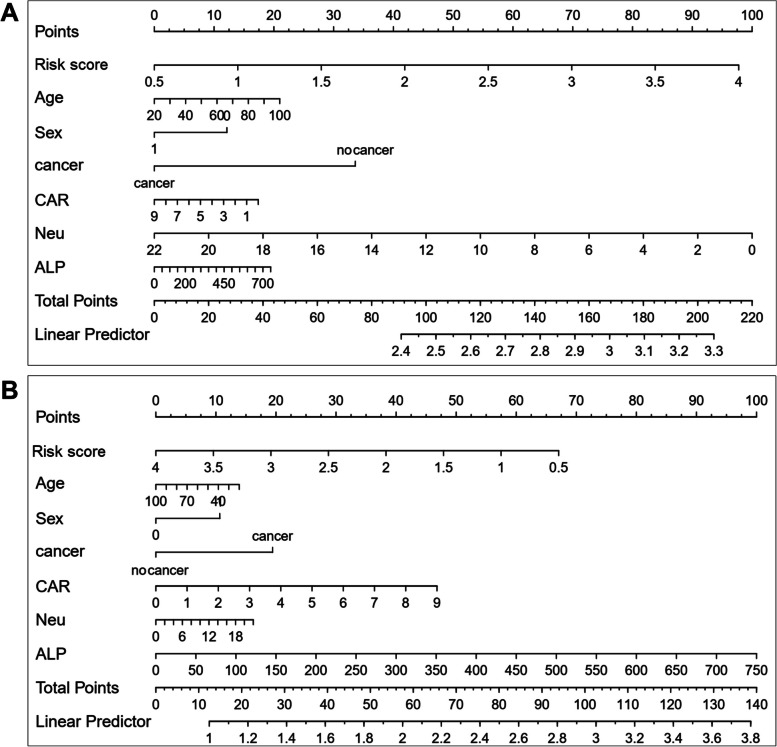
Nomogram of the **A** decompensated cirrhotic patients; **B** decompensated cirrhotic patients with SBP only

Next, a Cox regression analysis was applied to screen the risk factors associated with patient prognosis. As shown in Table 5, CAR, neutrophils and ALP were associated with the prognosis of decompensated patients with cirrhosis, while platelets, neutrophils, NLR, CAR and ALP were significantly associated with the prognosis of patients with SBP. These results suggested that CAR, neutrophils and ALP could predict the prognosis of decompensated cirrhotic patients with or without SBP. Finally, we used CAR, neutrophils and ALP to construct a prognostic model for decompensated cirrhotic patients and calculated the risk score from the Cox regression model and visualized it by a nomogram. We found that the risk score calculated from neutrophils, ALP and CAR showed a much better prognostic value in decompensated patients with cirrhosis than other indices, regardless of the decompensated patients with or without SBP (Fig. 4).

**Table 5:** Cox regression analysis for the risk factors related to the prognosis of patients

Features	Cirrhotic patients		SBP only	
	HR (univariable)	*P* value	HR (univariable)	
ALB	0.97 (0.90–1.05)	0.451	0.98 (0.96–1.00)	0.055
NLR	1.01 (0.99–1.03)	0.237	1.01 (1.00–1.03)	0.021
CAR	1.23 (1.08–1.41)	0.002	1.16 (1.08–1.24)	< 0.001
Platelet	1.00 (1.00–1.01)	0.656	1.00 (1.00–1.00)	0.001
ALT	1.00 (1.00–1.00)	0.910	1.00 (1.00–1.00)	0.138
Neutrophils	1.09 (1.03–1.16)	0.005	1.10 (1.06–1.14)	< 0.001
ALP	1.01 (1.00–1.01)	0.016	1.00 (1.00–1.00)	0.001
FNR	1.00 (1.00–1.00)	0.309	1.00 (1.00–1.00)	0.849

## Discussion

In the present study, we retrospectively analyzed data from 413 cirrhosis patients and found that 58 (11.4%) patients with SBP experienced complications, a prevalence consistent with previous reports [3, 4]. We found that SBP occured in a considerable number of patients with decompensated cirrhosis, suggesting that the progression of the disease was significantly associated with SBP. We also found that cirrhotic patients with cancer were more prone to develop SBP compared to non-cancer patients. The results of the laboratory test indices showed that most of the inflammatory indices, liver function indices and nutritional indices were significantly different between patients with and without SBP. Importantly, we discovered that cancer had differential effects on various laboratory test indices in cirrhotic patients without SBP, but had little effect in patients with SBP. Next, we examined the laboratory test indices associated with SBP in decompensated cirrhotic patients using two machine learning methods and validated by a logistic regression model. The results showed that ALB, NLR and FNR were independently associated with the SBP in which FNR had not previously been reported. Finally, we identified neutrophils, ALP, and CAR, all of which were associated with survival in cirrhotic patients with or without SBP, and the prognostic value constructed by these three indices was high compared to other indices. Taken together, these results demonstrated that several indices were associated with the occurrence of SBP in decompensated cirrhotic patients and FNR was a novel biomarker for the diagnosis of cirrhosis with SBP. We also identified three indicators that were strongly linked to the prognosis of decompensated cirrhotic patients.

SBP is a common complication in decompensated cirrhotic patients. Currently, the diagnosis of SBP in cirrhotic patients relies on testing for ascites, and abdominal paracentesis is an invasive procedure that increases the risk of infection [18]. As a result, it is beneficial for cirrhotic patients to develop a non-invasive method for diagnosing SBP. Using laboratory test indices from blood samples to diagnose SBP or estimate survival in cirrhotic patients is a viable approach in clinical practice. Previous studies have identified several common blood indices related to the SBP in cirrhosis, including CRP and NLR [11]. In the present study, we confirmed the diagnostic value of NLR in SBP of cirrhosis, indicating the robustness of this index. More importantly, as the diagnostic value of FNR in SBP in cirrhosis was reported for the first time, these results underscored the role of FNR in decompensated patients with cirrhosis.

Serum ferritin reflects the body's iron stores, which increases in iron overload and decreases in patients with iron deficiency disorders. Elevated liver iron would promote increased ferritin synthesis [19], and ferritin could activate the production of collagen and liver fibrogenesis [20]. Therefore, serum ferritin could be a useful marker of ongoing fibrosis [21]. In this study, we found that serum ferritin levels were significantly different between cirrhotic patients with and without SBP, but as shown in Table 3, the change in ferritin levels was strongly influenced by cancer, suggesting that the change in ferritin in cirrhotic patients with SBP were affected by cancer, so it could not be an independent index to diagnose SBP in cirrhosis. We therefore employed ferritin in conjunction with other blood indicators. One study reported that FAR was able to determine mortality in critically ill COVID-19 patients treated in the ICU [22]. Our study showed that FAR was elevated in decompensated cirrhotic patients and patients with SBP, however, it was not an independent index of SBP and was not associated with survival of cirrhotic patients.

FNR was estimated using the ratio of ferritin and neutrophils. The neutrophil count is a sensitive parameter of inflammation in the body. The combination of neutrophils with other indices has proven critical in diagnosing or predicting multiple diseases such as platelets to neutrophils ratio in a stroke [23], eosinophil-neutrophil ratio in tumors [24], platelet-to-neutrophil ratio in lupus nephritis [25]. This evidence showed that the combination of neutrophils with other indices had a more important value than the individual index. This study found that FNR was independently associated with the risk of SBP and worsened in patients with cirrhosis. We hypothesized that the mechanism underlying the value of FNR in cirrhotic patients with SBP might be that ferritin reflected the status of cirrhosis while neutrophils reflected infection of the peritoneum, and this combination might reflect the overall status of cirrhotic patients with SBP better.

Regarding the prognostic value of laboratory test indices associated with cirrhotic patients, one study reported that serum CRP levels were associated with a higher mortality rate in patients with SBP [26]. Ascitic fluid lactate and NLR [27], and calprotectin [28] were also associated with the mortality of patients with SBP. The present study showed that blood neutrophils, ALP and CAR levels were significantly associated with the prognosis of decompensated cirrhotic patients regardless of the decompensated patients with or without SBP, and the prognostic value of the model constructed by these indices was better than the individual index. These results indicated that the combination of certain laboratory tests indices could achieve a better prognostic value in cirrhotic patients.

There were several limitations in this study. First, although we included a large sample of cirrhotic patients, the number of SBP was relatively small, hence the survival analysis for cirrhotic patients with SBP could not be performed. Second, since this study is retrospective, the chosen bias is inevitable and confounding factors could undermine the robustness of the results. Third, this study is a single center research, so our results should be interpreted with caution when extrapolating to other population regions. Fourth, some patients with SBP didn’t accept diagnostic abdominal paracentesis and their ascitic fluid characteristics were unavailable, we could not further investigate the correlation of the biomarkers in ascitic fluid with SBP. Fifth, though the diagnosis model showed a great discrimination with the AUC of 0.808, its low false positive rate and high false negative rate indicated that it was best employed as an exclusive diagnosis tool. Therefore, a prospective multi-center design cohort is warranted to validate our results.

## Conclusions

The present study systematically analyzed the risk factors associated with the occurrence and prognosis of SBP in decompensated cirrhotic patients and identified FNR as a new index for the diagnosis of SBP in decompensated cirrhotic patients. A model constructed from neutrophils, ALP and CAR showed high performance in predicting the prognosis of patients with decompensated cirrhosis.

### Supplementary Information

**Additional file 1: Supplementary Table S1. **The laboratory indicators of ascites and serum PCT in 52 patients with SBP. **Supplementary Table S2.** The clinical characteristics of 12 patients with PMN < 250×106/L and PCT≤0.5 ng/ml.

## Data Availability

The data used to support the findings of this study are available from the corresponding author upon reasonable request.
