# Supplementary material for: Systematically analysis of decompensated cirrhotic patients with spontaneous bacterial peritonitis to identify diagnostic and prognostic indexes
Source: BMC Infect Dis. 2023 Nov 11;23:786. doi: 10.1186/s12879-023-08731-w (PMC10640738; doi:10.1186/s12879-023-08731-w)
Supplement: Supplementary file 1 — Additional file 1: Supplementary Table S1. The laboratory indicators of ascites and serum PCT in 52 patients with SBP. Supplementary Table S2. The clinical characteristics of 12 patients with PMN < 250×106/L and PCT≤0.5 ng/ml. [file 12879_2023_8731_MOESM1_ESM.pdf]

Supplementary Table S1. The laboratory indicators of ascites and serum PCT in 52 patients with SBP

Patient	Paracentesis	Ascites						Serum
		Karyocyte counts (×10^6/L)	PMC counts (×10^6/L)	PMN counts (×10^6/L)	MN counts (×10^6/L)	Alb	LDH (U/L)	PCT (ng/mL)
1	Yes	800	40	200	560	2.9	983.8	
2	No							0.775
3	No							
4	Yes							0.936
5	No							1.2
6	No							0.487
7	Yes	2100	63	1092	945	7.2	4.8	0.09
8	Yes	456	159.6	91.2	205.2	4.6	6.5	0.546
9	Yes	150	15	82.5	52.5	4.8	4.3	2.12
10	Yes	1200	24	1080	96	6.9	8.2	
11	No							0.103
12	Yes	300	30	45	225	5.1	9.1	0.274
13	Yes	1500	150	300	1050	6.7	3.7	
14	Yes	1197	0	143.64	1053.36	22.1	19.9	0.671
15	No							1.14
16	Yes	840	470.4	201.6	168	20		0.469
17	No							1.07
18	Yes	450	58.5	72	319.5	11.7	2.6	
19	No							0.495
20	No							1.07
21	No							1.39
22	Yes	40000	800	35200	4000	3.3	8	
23	Yes	344	17.2	154.8	172	6.6	5	0.354
24	Yes	860	43	602	215	5.5	6	1.04
25	Yes	324	0	97.2	226.8	3.5	7.8	0.237
26	Yes	3260	32.6	2999.2	228.2	4.6	4.2	0.201
27	Yes	250	25	50	175	13.8	14.8	
28	No							
29	No							0.561
30	Yes	156	18.72	85.8	51.48	15.4	4.5	0.267
31	Yes	150	15	105	30	3.4	7.2	0.897
32	No							0.523
33	Yes	2160	0	1728	432	13.2	6.1	0.78
34	Yes	2826	0	1045.62	1780.38	16.3	7.8	0.649
35	Yes	180	9	45	126			1.1
36	Yes	756	151.2	37.8	567	11.1	6.1	0.094
37	Yes	800	0	240	560	13.6	7.5	
38	No							0.691

39	Yes	200	30	100	70	6.9	8.3	0.068
40	Yes	200	20	120	60	1.4	3.5	0.472
41	No							0.678
42	Yes	400	0	260	140			
43	No							0.54
44	Yes	210	10.5	52.5	147	4.7	5.5	0.172
45	Yes	1620	81	972	567	23.4	9.7	1.32
46	Yes	60	3	18	39	1.8	2.2	2.11
47	No							6.39
48	Yes	5994	0	4795.2	1198.8	15.6	7.82	6.82
49	Yes	1000	40	780	180	2.3	2.8	5.99
50	Yes	2400	0	960	1440	3.5	5.2	0.272
51	No							
52	No							1.75

PMC= Peritoneal mesothelial cell; PMN= Polymorphonuclear leukocyte; MN= Monocyte; Alb= Albumin; LDH= Lactate dehydrogenase; PCT= Procalcitonin.

Supplementary Table S2. The clinical characteristics of 12 patients with PMN < 250×10^6/L and PCT≤0.5 ng/ml

Patients (n=12)	PMN<250×10^6/L and PCT≤0.5 ng/ml with following symptom
6	Obvious abdominal pain, abdominal tenderness, or rebound tenderness.
1	Abdominal tenderness and intestinal obstruction.
2	Fever with systemic inflammatory response syndrome and deterioration of the liver and kidney function.
3	hepatic encephalopathy without inducement.
1	no response to diuretics and had an acute kidney injury.
